# Role of Transportation in Spread of Porcine Epidemic Diarrhea Virus Infection, United States

**DOI:** 10.3201/eid2005.131628

**Published:** 2014-05

**Authors:** James Lowe, Phillip Gauger, Karen Harmon, Jianqiang Zhang, Joseph Connor, Paul Yeske, Timothy Loula, Ian Levis, Luc Dufresne, Rodger Main

**Affiliations:** University of Illinois, Urbana, Illinois, USA (J. Lowe);; Iowa State University, Ames, Iowa, USA (P. Gauger, K Harmon, J. Zhang, R. Main);; Carthage Veterinary Service, Ltd., Carthage, Illinois, USA (J. Connor);; Swine Vet Center, P.A., St. Peter, MN, USA (P. Yeske, T. Loula);; Iowa Select Farms, Iowa Falls, Iowa, USA (I. Levis);; Seaboard Foods, Kansas City, Missouri, USA (L. Dufresne)

**Keywords:** Porcine Epidemic Diarrhea Virus, PEDV, viruses, *Cornaviridae*, transport, lairage, biosecurity, epidemic, fomite

## Abstract

After porcine epidemic diarrhea virus (PEDV) was detected in the United States in 2013, we tested environmental samples from trailers in which pigs had been transported. PEDV was found in 5.2% of trailers not contaminated at arrival, , suggesting that the transport process is a source of transmission if adequate hygiene measures are not implemented.

Porcine epidemic diarrhea virus (PEDV) was detected in herds of pigs in the United States during April 2013 (*1*). PEDV is a member of the *Cornaviridae* family that produces a malabsorptive diarrhea secondary to atrophy of the small intestinal villi (*2*). Initial clinical cases were detected in herds in Indiana and Iowa during May 2013.The virus spread rapidly across large geographic regions; 218 cases of infection were identified in 16 states during the first 9 weeks of the outbreak (*3*). Subsequent testing of historical samples collected during the week of April 15, 2013 identified the index herd in Ohio (*3*). Veterinarians became concerned about the role that facilities where pigs are harvested for processing into food and the transportation equipment used to move pigs from farms to those facilities were playing in the spread of PEDV. These concerns were based on evidence that equipment used to transport live pigs transmits another enteric coronavirus, transmissible gastroenteritis virus, between sites in the United States (J.F. Lowe, unpub. data).

Pigs are commonly transported to harvest facilities in vehicles that have not been cleaned and disinfected between loads. Implementation of “all in–all out” sites, which are sites in which pigs are grown and all pigs in a group are removed before arrival of the next group, limits the spread of disease introduced by transport vehicles. In many cases, the risks and associated costs of disease introduced late in the growing period are thought to be less than the cost of cleaning and disinfecting vehicles. Transport vehicles are often shared by different pig owners, enabling the spread of disease across large regions.

## The Study

The objective of this study was to assess the risks that harvest facilities and transport vehicles engendered in promoting the initial outbreak of a novel disease organism by estimating the incidence of trailer contamination with PEDV during the unloading process at harvest facilities. Environmental samples were collected from 575 livestock trailers before and after pigs were unloaded into holding pens, or lairages, at 6 harvest facilities (83–102 trailers per facility) located in the central United States. Samples were collected during a period of 2–3 days at each facility during June 14–20, 2013. For each trailer, the following information was collected: transport company and trailer identification, time of unloading, dock used, whether the truck driver stepped on the dock, and whether facility personnel entered the trailer. Sample collection consisted of rubbing a phosphate-buffered saline–moistened pad (Swiffer, Procter & Gamble, Cincinnati, OH, USA) over an ≈900 cm^2^ area of the trailer floor, 15 cm from the rear door. The pad was placed in a sterile bag (Whirl-Pac, NASCO, Fort Atkinson, WI, USA) and the liquid was collected by applying manual pressure. The liquid was transferred to a sterile tube (14mL Falcon Tube, Fisher Scientific, Chicago, IL, USA), immediately placed on ice, and maintained at 4°C during transport to the Iowa State University Veterinary diagnostic laboratory. New latex gloves were worn for each sample collection to minimize the risk for cross-contamination.

RNA extraction was performed with 100 μL of each environmental sample by using the MagMAX Viral RNA Isolation Kit (Life Technologies, Carlsbad, CA, USA) and a Kingfisher 96 instrument (Thermo Scientific, Waltham, MA, USA) and Kingfisher program AM_1836_DW_HV_v3 provided by the manufacturer of the viral extraction kits. Viral RNA was eluted into 90 μL of buffer. Real-time reverse transcription PCR (rRT-PCR) was performed on nucleic acid extracts by using the Path-ID Multiplex One-Step RT-PCR Kit (Life Technologies) according to the manufacturer’s recommendations. Primers and probe targeting conserved regions of the PEDV nucleocapsid protein gene were as described (*4*) with modifications specific to the sequence isolated in North America deposited in GenBank (accession no. KF272920). The forward primer sequence was 5′-CGCAAAGACTGAACCCACTAACCT-3′, the reverse primer sequence was 5′-TTGCCTCTGTTGTTACTTGGAGAT-3′, and the probe sequence was 5′-TGTTGCCATTACACCGACTCCTGC-3′. Sequences were labeled by using the FAM/ZEN/3′ Iowa Black detector (Integrated DNA Technologies, Coralville, IA, USA). All rRT-PCR reactions were conducted on an ABI 7500 Fast (Applied Biosystems, Foster City, CA, USA) and results analyzed by system software. Samples were tested separately from routine diagnostic samples in the laboratory to minimize risks for cross-contamination.

Before unloading, 38 (6.6%) of the 575 trailers were contaminated with PEDV. The proportion of contaminated trailers ranged from 2% to 14.6% among the 6 harvest facilities; the facility level median was 5.0%. Of the remaining 537, 28 (5.2%) that were not contaminated at arrival were contaminated in the unloading process (Table).

**Table:** Status of environmental samples from pig transport trailers during an outbreak of porcine epidemic diarrhea virus infection, Midwestern United States, June 2013*

PCR status after unloading	PCR test status before unloading		
	Positive	Negative	Total
Positive	25 (4.3)	28 (4.9)	53 (9.2)
Negative	13 (2.3)	509 (88.5)	522 (90.8)
Total	38 (6.6)	537 (93.4)	575

*Values represent the number of trailers (% total) in each group. Samples were gathered from 6 pig harvest facilities.

Of the 38 trailers that were contaminated on arrival, environmental samples from 13 (34.2%) were negative for PEDV after unloading. Environmental samples from these 13 trailers tended to have higher cycle threshold values than those from the 25 trailers that were positive before and after unloading: 32.3 versus 30.6, respectively. This result suggests that the pigs transported to the harvest facility on the 13 trailers may not have been shedding PEDV, but instead, the trailers had been contaminated by previous loads of pigs, so viral quantities in the trailer were low or at the limit of detection.

Contamination during unloading occurred at a higher rate if harvest facility staff entered the trailer (OR 4.15, 95% CI 1.27–13.54) or if unloading occurred immediately after unloading another trailer that was found to be contaminated (OR 3.35, 95% CI 1.22–9.18). Facilities in which more PEDV was identified in truck trailers on arrival had a higher overall incidence of contamination. This was measured by multiplying the prevalence of contamination at arrival by the inverse of the cycle threshold value from trailers contaminated at arrival (R^2^ = 0.32, p = 0.01). All drivers stepped into the harvest facility at least once, leading to a high rate of contact between drivers, the trailers, and the harvest facility.

## Conclusions

Harvest facilities serve as a source of contact between many swine farms with different health statuses. This study suggests that collection points, such as harvest facilities and livestock auction markets, can be an efficient source of contamination of transport vehicles that return to pig farms and likely played a role in rapidly disseminating PEDV across vast geographic regions shortly after PEDV was first identified in the United States. These data also suggest that the contamination of transport vehicles leaving the harvest facilities increased as the prevalence of PEDV–positive transport vehicles and virus load coming into the facility increased.

The results of this study suggest that proactive disease control measures should include improved sanitation, hygiene, and segregation practices at collection points to limit the spread of the agent early in the outbreak. Current data suggest that novel agents, such as PEDV, may be present in a country but remain undetected for an extended period. Thus, control measures may be implemented too late to limit the spread of the disease through fomites that are identified, such as, in this instance, contaminated vehicles returning from swine collection points. Simple measures such as limiting contact between drivers and the collection point and requiring drivers to remain on trucks and out of the collection point during the unloading process may have a dramatic effect on limiting the transmission of novel agents. These biosecurity measures are simple but require a coordinated effort between producers, transporters, harvest facility owners, and regulators to achieve effective implementation. This study of PEDV transmission by fomites should serve as an example of the risks that a modern, highly technical animal protein industry may encounter during a novel disease introduction. PEDV’s introduction and subsequent spread in the United States should spur action to minimize these risks before a disease that can affect international trade or food safety is introduced.
